# A comparative study of antihypertensive drugs prediction models for the elderly based on machine learning algorithms

**DOI:** 10.3389/fcvm.2022.1056263

**Published:** 2022-12-01

**Authors:** Tiantian Wang, Yongjie Yan, Shoushu Xiang, Juntao Tan, Chen Yang, Wenlong Zhao

**Affiliations:** ^1^School of Medical Informatics, Chongqing Medical University, Chongqing, China; ^2^Medical Records and Statistics Office, The Third Affiliated Hospital of Army Medical University, Chongqing, China; ^3^Medical Records and Statistics Room, Affiliated Banan Hospital of Chongqing Medical University, Chongqing, China; ^4^Operation Management Office, Affiliated Banan Hospital of Chongqing Medical University, Chongqing, China

**Keywords:** elderly hypertension, ML, LightGBM, personalized medicine, antihypertensive drug

## Abstract

**Background:**

Globally, blood pressure management strategies were ineffective, and a low percentage of patients receiving hypertension treatment had their blood pressure controlled. In this study, we aimed to build a medication prediction model by correlating patient attributes with medications to help physicians quickly and rationally match appropriate medications.

**Methods:**

We collected clinical data from elderly hypertensive patients during hospitalization and combined statistical methods and machine learning (ML) algorithms to filter out typical indicators. We constructed five ML models to evaluate all datasets using 5-fold cross-validation. Include random forest (RF), support vector machine (SVM), light gradient boosting machine (LightGBM), artificial neural network (ANN), and naive Bayes (NB) models. And the performance of the models was evaluated using the micro-F1 score.

**Results:**

Our experiments showed that by statistical methods and ML algorithms for feature selection, we finally selected Age, SBP, DBP, Lymph, RBC, HCT, MCHC, PLT, AST, TBIL, Cr, UA, Urea, K, Na, Ga, TP, GLU, TC, TG, γ-GT, Gender, HTN CAD, and RI as feature metrics of the models. LightGBM had the best prediction performance with the micro-F1 of 78.45%, which was higher than the other four models.

**Conclusion:**

LightGBM model has good results in predicting antihypertensive medication regimens, and the model can be beneficial in improving the personalization of hypertension treatment.

## Introduction

Hypertension is one of the most common chronic diseases and a significant risk factor for cardiovascular disease, chronic kidney disease, cognitive impairment, all-cause mortality, and disability (1). Therefore, the elderly population is a priority population of concern. The relationship between the elderly and the prevalence of hypertension has been demonstrated in studies in various countries (2–6). From 1976 to 2017, the majority of hypertension among women and men aged 60 years or older fluctuated from 60 to 75% in a 123-item national health screening survey of 12 high-income nations and reached this high level in the early 2000s and has been reasonably consistent since then (7). The same trend was found in the seventh report of the Joint National Committee (JNC) on Prevention, Detection, Evaluation, and Treatment of Hypertension, with more than 65% of older adults suffering from hypertension (8). At the same time, older patients have a lower control rate than younger patients in hypertension (9). With the acceleration of population aging, the number of elderly hypertension patients is also increasing yearly, causing a heavy burden on the world's public health.

Maintaining appropriate blood pressure is a critical strategy for lowering the incidence of cardiovascular illness, disability, and mortality. Nowadays, the treatment options for hypertension mainly include life interventions and pharmacological treatment. The common antihypertensive drugs can be divided into five categories: angiotensin-converting enzyme inhibitors (ACEI), angiotensin II receptor antagonists (ARB), calcium channel blockers (CCB), β-blockers, and diuretics. According to the biggest research ever conducted on hypertension, between 1990 and 2019, fewer than one-quarter of hypertensive women and one-fifth of hypertensive men could regulate their blood pressure to normal levels with medication (10). The World Health Organization (WHO) and the International Society of Hypertension (ISH) recommended individualized regimens for the treatment of patients with hypertension (11). The JNC recommended employing 12-lead ECG, urinalysis, blood glucose, hematocrit, serum potassium, creatinine, calcium levels, and fasting lipoprotein profiles, which included high-density lipoprotein cholesterol, low-density lipoprotein cholesterol, and triglycerides (8). The importance of individualizing hypertension treatment is further illustrated.

Electronic Health Record (EHR) is the digitization of an individual's health record. It contains patient data collected during clinical care, including diagnostic billing codes, procedure codes, vital signs, laboratory test results, clinical imaging, and physician records. In recent years, the EHR has become a centerpiece of hospital information systems as healthcare information technology continues to evolve and the demand for clinical and record information management surges. EHR data are used in various applications, including epidemiological and observational studies, safety surveillance and regulatory use, and prospective clinical studies. Examples: Tabesh et al. (12) analyzed shifts in antihypertensive and lipid-lowering medication therapy in individuals with type 2 diabetes between 2006 and 2015 using EHR data. The US Food and Drug Administration (FDA) conducted post-market safety investigations using EHR data from various sources (13). Jernberg et al. (14) used EHR data to improve care and develop treatments for coronary artery disease based on scientific evidence. According to Cowie (15), EHR should improve clinical diagnosis.

The research on ML of hypertension involves the construction of hypertension risk prediction models (16, 17) as well as the risk prediction research of hypertension complicated by other diseases (18, 19). Recently, Gideon et al. applied ML techniques like decision tree and neural network to find out how to treat hypertension in a large group of Maccabi Health Service patients in Israel (20). Their analysis revealed that 17,234 people were treated with a single medicine, much more than the number of patients treated with a combination treatment. The effectiveness of monotherapy was around 44%. Although this success rate was unsatisfactory, it was still more than the success rate of two, three, or four medication combinations. Liu et al. (21) plotted the profile of five widely used antihypertensive medications (Irbesartan, Metoprolol, Felodipine, Amlodipine, Levamlodipine). Cr and Cys were the common crucial factors affecting its drug control rate. In addition, K^+^, FPG, and LDL also impacted Irbesartan; Metoprolol was affected by Age, Urea, and HCT; Felodipine was affected by FPG, Age, and HB; and Amlodipine was affected by FPG, Age, and Urea. Liu et al. (22) found that HCT, HB, CR, Urea, CYS, age, sex, SBP, DBP, PP, and HR were significant and sensitive factors influencing the rate of hypertension management with Metoprolol. These studies might help tailor the administration of effective antihypertensive drugs to the individual patient, but no final models accounting for these variables were built. Our study used EMR data to describe drug-related features from a vast data set. Then, we constructed medication prediction algorithms to optimize individualized medicine usage and give a reference for physicians.

## Materials and methods

### Data sources and population

The data we collected comes from the MeduCloud data platform of Chongqing Medical University. It brings together 40 million EMR data from seven general hospitals in the western city of Chongqing, China, containing basic patient information, admission records, medical course records, test and examinations, symptoms and signs, and medication information. The study did not involve human or animal participants, and all data were identified before collection to comply with ethical requirements.

Data were included from 2016.12 to 2021.12 for inpatients aged ≥65 years who had registered antihypertensive drugs in the medication information and whose diagnosis of “primary hypertension” was recorded in the discharge report. Although the MeduCloud data platform documented the antihypertensive medicine usage of many outpatients, there was insufficient clinical test data to match the requirements of this research; thus, only inpatients were included.

### Data extraction

We chose gender and age at the time of presentation from basic information, blood pressure from admission records, and a history of hypertension (HTN), coronary artery disease (CAD), diabetes, stroke, and renal insufficiency (RI). In the beginning, 47 indicators were chosen from the clinical tests based on the recommendations of specialists. However, certainly expected indications were absent from the majority of samples. In addition to the fact that professionals pay more attention to the essential characteristics during real diagnosis and therapy, we ultimately chose 22 attributes. The 22 attributes were: glucose (GLU), total cholesterol (TC), triglycerides (TG), potassium (K), sodium (Na), calcium (Ca), creatinine (Crea), serum uric acid (UA), serum urea (Urea), aspartate aminotransferase (AST), alanine aminotransferase (ALT), γ-glutamyl transpeptidase (γ-GT), total bilirubin (TBIL), total protein (TP), red blood cell (RBC), hematocrit (HCT) level, mean corpuscular volume (MCV), mean corpuscular hemoglobin (MCH), mean corpuscular hemoglobin concentration (MCHC), lymphocyte (Lymph) count, platelet (PLT) count, and mean platelet volume (MPV). The standard reference ranges for blood pressure and laboratory indices were listed in Supplementary Table S1.

### Statistical analysis

We used SPSS 26.0 and Excel 2013 software for statistical analysis. Kolmogorov-Smirnov was used to test data normality. x ± s expressed the normal distribution, and the comparison between groups was performed by analysis of variance. The non-normal distribution data were described as M (Q1, Q3), and the Kruskal–Wallis test was used to compare groups. The categorical indicators were expressed by rate (%), and the χ^2^-test was used to compare groups. *P* < 0.05 was considered a statistically significant difference. For the algorithm, we relied on Python (version 3.97).

### Machine learning algorithms

In this study, we used five ML algorithms for feature selection, namely Pearson correlation coefficient, Lasso, maximum information coefficient (MIC), random forest (RF), and recursive feature elimination (REF). The five ML models were built into an ensemble model according to specific rules. Finally, combined the ensemble model with statistical analysis to obtain the final feature indicators. For a better comparison, values of all attributes were Min-Max normalized to a standard range using the following formula:

Normalized value = (original value – minimized value) / (maximized value – minimized value)

We compared the performance of RF, SVM, LightGBM, ANN, and NB, which are all well-known ML classification algorithms, to find the best model for predicting medication use. To segment the data for each algorithm, we used a 5-fold cross-validation approach, trained the model through the training set each time, and validated the model's performance on the test set data. Due to the imbalance between the three predicted medication classes in this study, we used micro-F1 to assess the model's efficacy. Higher values of micro-F1 indicated better model performance, and it can be used for both multi-class classification problems and asymmetrical data. The calculation is as follows (for three categories):


Recallm=TP1+TP2+TP3TP1+TP2+TP3+FN1+FN2+FN3Precisionm=TP1+TP2+TP3TP1+TP2+TP3+FP1+FP2+FP3                            micro−F1=2Re callm×PrecisionmRe callm+Precisionm

*TP*i refers to a true positive of class i; *FP*i refers to a false positive of class i; *TN*i refers to a true negative of class i; *FN*i refers to a false negative of class i.

**Figure 1:**
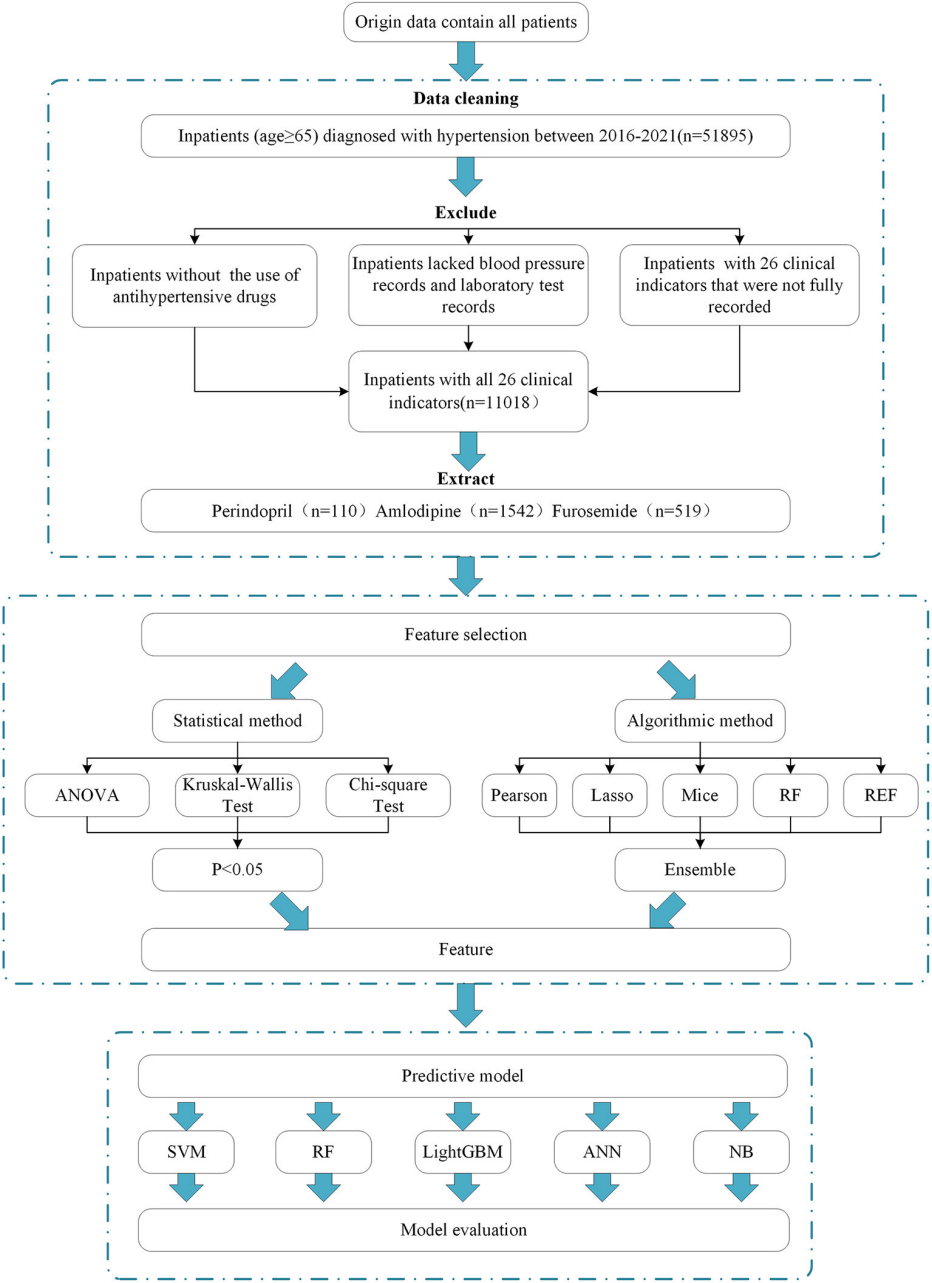
The flow chart of the data process and analysis.

The flow chart of the data process and analysis is shown in Figure 1.

## Results

### Brief introduction of the cases selected for the study

**Figure 2:**
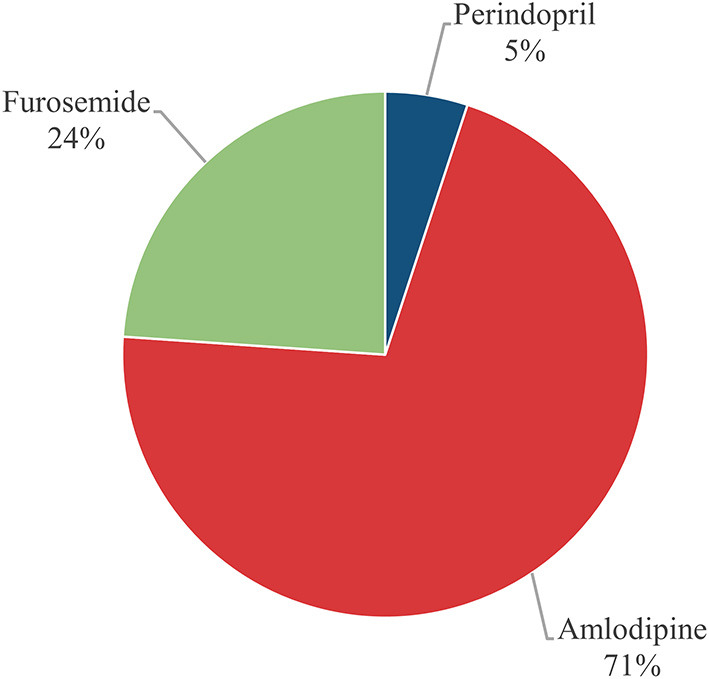
The proportion of the three antihypertensive drugs.

We looked up “2016.12-2021.12, age ≥65 years, primary hypertension” on the MeduCloud platform to get a sample of 51,895 cases. Firstly, we excluded 14,950 cases that did not register the use of antihypertensive drugs. Secondly, removed 10,884 cases lacked blood pressure and laboratory test records. Finally, deleted 15,043 cases that did not have complete records of 26 clinical indicators. After data cleaning, 11,018 cases were left. In light of the actual situation, we selected three more frequent drugs for studying. They were 110 cases of Perindopril, 1,542 cases of Amlodipine, and 519 cases of Furosemide (Figure 2). Meanwhile, we counted the number of patients (36,945) taking antihypertensive drugs (Supplementary Table S2, Supplementary Figure S1).

### Test the difference in the indexes in patients using different kinds of drugs

The results showed that there was a significant difference in age between the groups (H = 107.33, *P* = 0.000), and the age of patients in the group using Furosemide [79.00 (73.00, 84.00)] was higher than in the other two groups [74.00 (69.00, 79.00)]. In laboratory tests, there were significant differences in SBP, DBP, Lymph, RBC, HCT, MCHC, PLT, AST, TBIL, Cr, UA, Urea, K, Na, Ga, TP, GLU, TC, TG, and γ-GT (*P* < 0.05, Table 1). There was a significant difference in the proportion of males and females using drugs in the three groups (χ^2^ = 20.665, *P* = 0.000). Men use Amlodipine and Furosemide more frequently than women, and in contrast, women use Perindopril more regularly than men. Among previous disease history, there was a statistically significant difference in HTN (χ^2^ = 31.664, *P* = 0.000), CAD (χ^2^ = 24.805, *P* = 0.000), and RI (χ^2^ = 11.806, *P* = 0.003) (Table 1).

**Table 1:** Test the difference in the indexes in patients using different kinds of drugs.

**Variable**	**Perindopril**	**Amlodipine**	**Furosemide**	**c2/H/F**	***P***
Male [cases (%)]	46 (41.80)	883 (57.30)	274 (52.80)	20.67	<0.001
HTN [cases (%)]	61 (55.50)	1,156 (75.00)	419 (80.70)	31.66	<0.001
CAD [cases (%)]	16 (14.50)	273 (17.70)	142 (27.40)	24.81	<0.001
RI [cases (%)]	0 (0.00)	5 (0.30)	12 (2.30)	11.81	<0.001
Diabetes [cases (%)]	19 (17.30)	296 (19.20)	123 (23.70)	5.50	0.060
Stroke [cases (%)]	7 (6.40)	122 (7.90)	31 (6.00)	2.31	0.320
Age [year, M (Q1, Q3)]	74.00 (69.00, 79.00)	74.00 (69.00, 79.00)	79.00 (73.00, 84.00)	107.33	<0.001
SBP [mmHg, M (Q1, Q3)]	143.50 (128.50, 155.50)	144.00 (131.00, 158.00)	134.00 (120.00, 147.00)	102.81	<0.001
DBP [mmHg, M (Q1, Q3)]	81.00 (72.00, 89.25)	80.00 (72.00, 88.00)	74.00 (65.00, 84.00)	67.81	<0.001
Lymph [× 109/L, M (Q1, Q3)]	1.42 (1.05, 1.76)	1.41 (1.07, 1.80)	1.04 (0.73, 1.46)	158.85	<0.001
RBC [× 1012/L, M (Q1, Q3)]	4.33 (4.03, 4.71)	4.26 (3.91, 4.61)	3.83 (3.35, 4.30)	162.97	<0.001
HCT [%, M (Q1, Q3)]	40.15 (36.53, 43.23)	39.00 (36.10, 42.00)	35.00 (30.20, 39.40)	157.10	<0.001
MCV [fl, M (Q1, Q3)]	92.00 (89.45, 94.58)	92.10 (88.80, 95.10)	92.60 (88.60, 96.80)	5.29	0.070
MCH [pg, M (Q1, Q3)]	30.50 (29.50, 31.60)	30.50 (29.40, 31.60)	30.50 (28.80, 32.00)	0.11	0.950
MCHC [g/L, M (Q1, Q3)]	332.00 (324.75, 338.00)	332.00 (324.00, 339.00)	329.00 (319.00, 338.00)	14.58	<0.001
PLT [× 109/L, M (Q1, Q3)]	172.00 (127.75, 215.00)	189.00 (151.75, 232.00)	169.00 (128.00, 233.00)	31.16	<0.001
MPV [fl, M (Q1, Q3)]	11.10 (9.90, 12.30)	10.90 (10.00, 11.90)	10.80 (9.80, 11.80)	4.82	0.090
AST [U/L, M (Q1, Q3)]	20.00 (17.00, 25.00)	20.20 (16.8, 25.32)	23.00 (18.00, 38.00)	53.82	<0.001
ALT [U/L, M (Q1, Q3)]	16.00 (11.35, 22.00)	16.55 (11.90, 24.70)	17.00 (11.00, 30.50)	1.94	0.380
TBIL [μmol/, M (Q1, Q3)]	11.70 (9.18, 14.89)	10.30 (7.90, 13.45)	11.70 (7.90, 17.20)	27.45	<0.001
Cr [μmol/L, M (Q1, Q3)]	72.15 (58.80, 89.3)	67.39 (55.90, 81.93)	82.70 (62.60, 125.30)	101.52	<0.001
UA [μmol/L, M (Q1, Q3)]	323.35 (273.93, 412.05)	325.98 (259.00, 394.40)	372.50 (280.50, 485.60)	58.77	<0.001
Urea [mmol/L, M (Q1, Q3)]	5.77 (5.05, 7.39)	5.68 (4.61, 7.13)	7.33 (5.50, 11.07)	148.58	<0.001
K [mmol/L, M (Q1, Q3)]	4.03 (3.77, 4.24)	3.93 (3.64, 4.20)	4.06 (3.66, 4.50)	30.89	<0.001
Na [mmol/L, M (Q1, Q3)]	141.40 (139.78, 143.13)	141.23 (139.00, 142.95)	139.50 (136.00, 142.80)	55.86	<0.001
Ca [mmol/L, M (Q1, Q3)]	2.25 (2.19, 2.35)	2.24 (2.14, 2.33)	2.19 (2.05, 2.30)	43.10	<0.001
TP [g/L, x ± s]	68.94 ± 6.87	68.90 ± 6.77	65.49 ± 8.28	20.99	<0.001
GLU [mmoL/L, M (Q1, Q3)]	5.71 (4.92, 7.05)	5.77 (5.03, 7.31)	6.30 (5.10, 8.36)	18.93	<0.001
TC [mmoL/L, M (Q1, Q3)]	4.19 (3.62, 4.90)	4.37 (3.66, 5.10)	3.70 (2.92, 4.58)	123.77	<0.001
TG [mmoL/L, M (Q1, Q3)]	1.31 (0.97, 1.87)	1.29 (0.95, 1.75)	1.09 (0.80, 1.51)	42.07	<0.001
r-GT [U/L, M (Q1, Q3)]	21.78 (15.84, 28.02)	23.00 (16.68, 36.60)	33.00 (18.00, 63.00)	59.89	<0.001

### ML feature selection

The purpose of feature selection is to pick a set number of feature subsets that provide the least generalization error on the original sample or to decide the smallest feature subset possible under a given generalization error (23). Applying the concept of integration learning, we developed our own rules to combine the outputs of the five algorithms:

1. We recorded the feature ranking obtained by each algorithm. The higher the ranking number, the more influential the feature was.
2. We found the average order of each feature under the five algorithms.
3. We calculated the weight corresponding to the ranking average.

**Figure 3:**
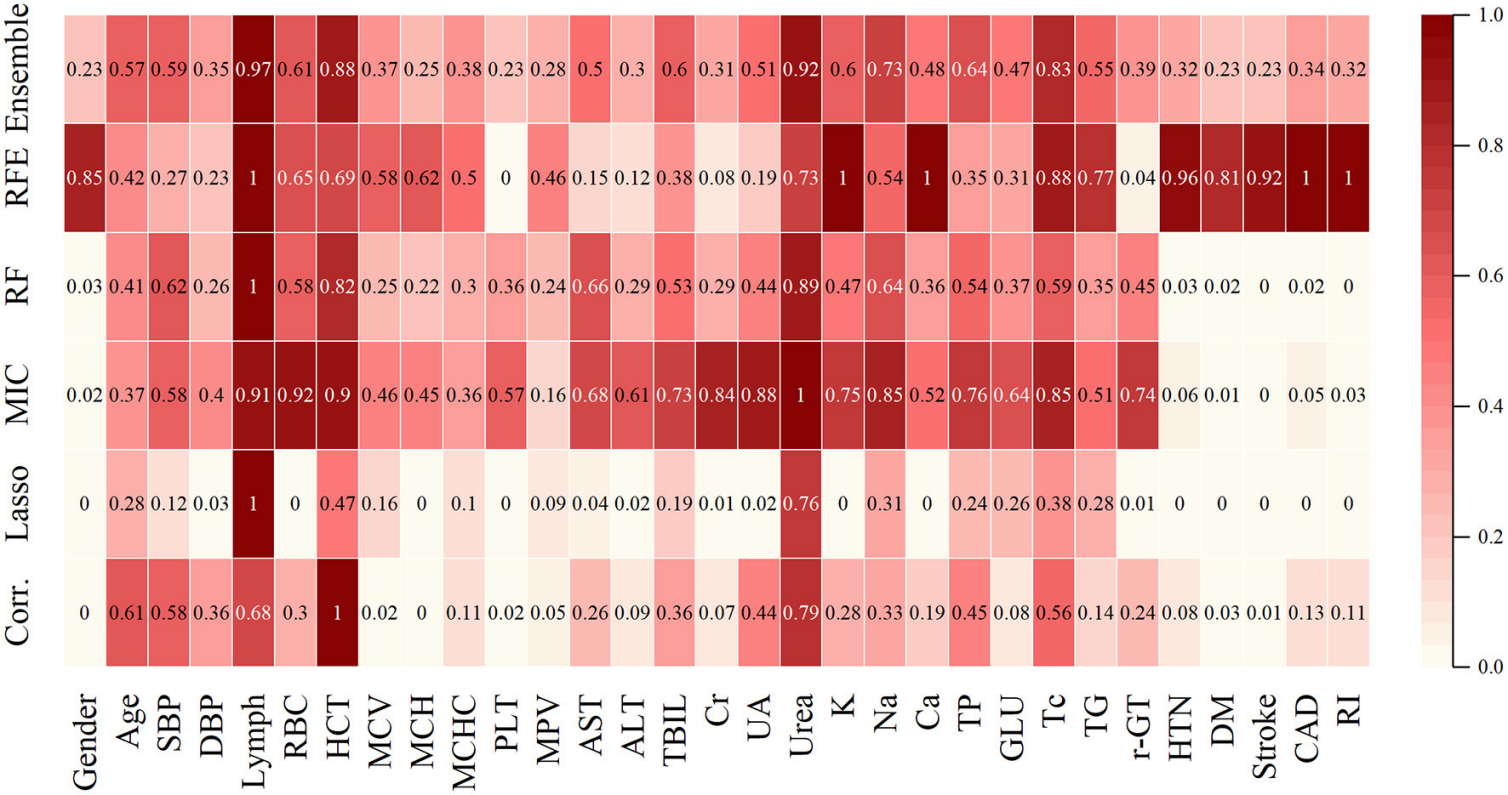
Different models calculated the weight of the features for the three drugs. The color represents the weight of the features, and their values are marked within the boxes.

According to the algorithm analysis, Lymph, Urea, HCT, TC, Na, TP, RBC, TBIL, K, SBP, Age, TG, UA, and AST were the essential features affecting the selection of medication for hypertension. The outcomes of the above feature selection procedures were summed together, with deeper colors indicating more crucial features (Table 2, Figure 3).

**Table 2:** Results of ML models for feature selection.

**Corr**.	**Weight**	**Lasso**	**Weight**	**MIC**	**Weight**	**RF**	**Weight**	**RFE**	**Weight**	**Ensemble**	**Weight**
HCT	1.00	Lymph	1.00	Urea	1.00	Lymph	1.00	Lymph	1.00	Lymph	0.97
Urea	0.79	Urea	0.76	RBC	0.92	Urea	0.89	K	1.00	Urea	0.92
Lymph	0.68	HCT	0.47	Lymph	0.91	HCT	0.82	Ca	1.00	HCT	0.88
Age	0.61	TC	0.38	HCT	0.90	AST	0.66	CAD	1.00	TC	0.83
SBP	0.58	Na	0.31	UA	0.88	Na	0.64	RI	1.00	Na	0.73
TC	0.56	Age	0.28	Na	0.85	SBP	0.62	HTN	0.96	TP	0.64
TP	0.45	TG	0.28	TC	0.85	TC	0.59	Stroke	0.92	RBC	0.61
UA	0.44	GLU	0.26	Cr	0.84	RBC	0.58	TC	0.88	TBIL	0.60
DBP	0.36	TP	0.24	TP	0.76	TP	0.54	Gender	0.85	K	0.60
TBIL	0.36	TBIL	0.19	K	0.75	TBIL	0.53	DM	0.81	SBP	0.59
Na	0.33	MCV	0.16	r-GT	0.74	K	0.47	TG	0.77	Age	0.57
RBC	0.30	SBP	0.12	TBIL	0.73	r-GT	0.45	Urea	0.73	TG	0.55
K	0.28	MCHC	0.10	AST	0.68	UA	0.44	HCT	0.69	UA	0.51
AST	0.26	MPV	0.09	GLU	0.64	Age	0.41	RBC	0.65	AST	0.50
r-GT	0.24	AST	0.04	ALT	0.61	GLU	0.37	MCH	0.62	Ca	0.48
Ca	0.19	DBP	0.03	SBP	0.58	PLT	0.36	MCV	0.58	GLU	0.47
TG	0.14	ALT	0.02	PLT	0.57	Ca	0.36	Na	0.54	r-GT	0.39
CAD	0.13	UA	0.02	Ca	0.52	TG	0.35	MCHC	0.50	MCHC	0.38
MCHC	0.11	Cr	0.01	TG	0.51	MCHC	0.30	MPV	0.46	MCV	0.37
RI	0.11	r-GT	0.01	MCV	0.46	ALT	0.29	Age	0.42	DBP	0.35
ALT	0.09	Gender	0.00	MCH	0.45	Cr	0.29	TBIL	0.38	CAD	0.34
GLU	0.08	RBC	0.00	DBP	0.40	DBP	0.26	TP	0.35	HTN	0.32
HTN	0.08	MCH	0.00	Age	0.37	MCV	0.25	GLU	0.31	RI	0.32
Cr	0.07	PLT	0.00	MCHC	0.36	MPV	0.24	SBP	0.27	Cr	0.31
MPV	0.05	K	0.00	MPV	0.16	MCH	0.22	DBP	0.23	ALT	0.30
DM	0.03	Ca	0.00	HTN	0.06	Gender	0.03	UA	0.19	MPV	0.28
MCV	0.02	HTN	0.00	CAD	0.05	HTN	0.03	AST	0.15	MCH	0.25
PLT	0.02	DM	0.00	RI	0.03	DM	0.02	ALT	0.12	Gender	0.23
Stroke	0.01	Stroke	0.00	Gender	0.02	CAD	0.02	Cr	0.08	PLT	0.23
Gender	0.00	CAD	0.00	DM	0.01	Stroke	0.00	r-GT	0.04	DM	0.23
MCH	0.00	RI	0.00	Stroke	0.00	RI	0.00	PLT	0.00	Stroke	0.23

### Combination of statistical analysis and algorithm results

**Figure 4:**
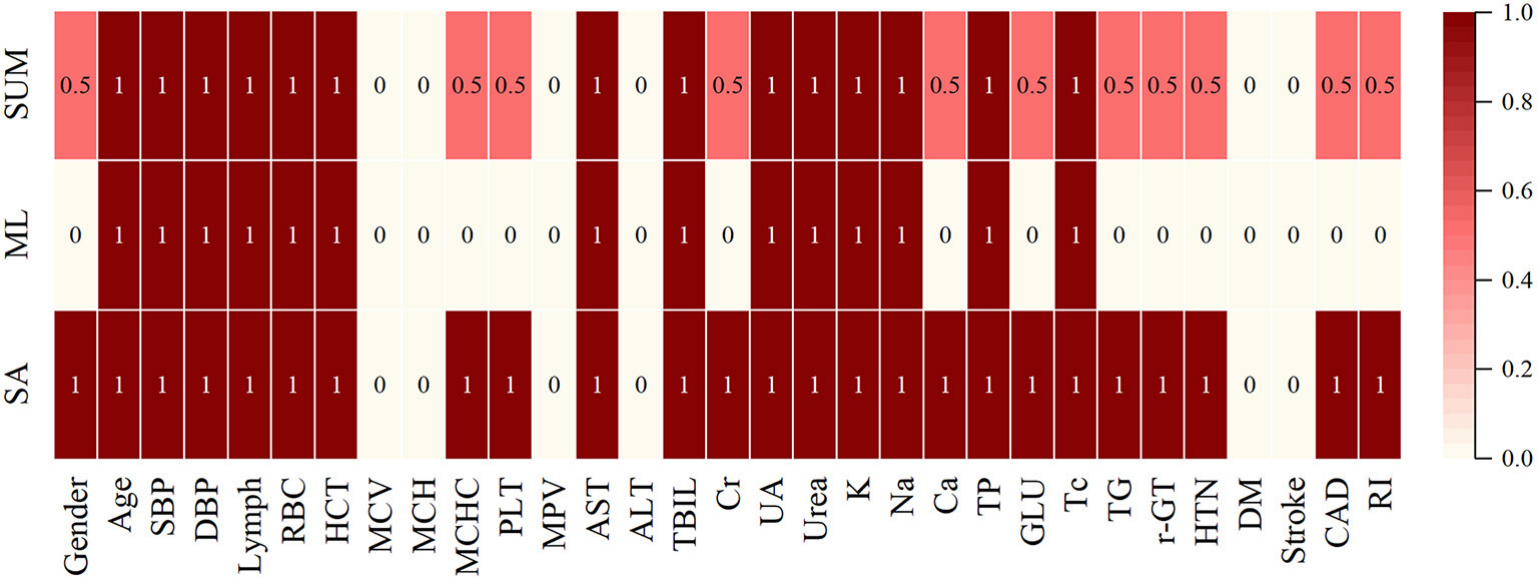
Final results of feature selection by data mining.

Combine statistical analysis with ML feature selection, where 1 (dark red) indicates that this feature is considered important by both statistics and algorithms, and 0 (light white) indicates that it is not regarded as relevant by either. If the algorithm does not value a feature, but the statistics do, we have a 0.5 (light red) feature, and vice versa (Figure 4).

In order not to omit the information related to the medication regimen, in the subsequent analysis, we still choose Age, SBP, DBP, Lymph, RBC, HCT, MCHC, PLT, AST, TBIL, Cr, UA, Urea, K, Na, Ga, TP, GLU, TC, TG, γ-GT, Gender, HTN, CAD, RI as feature indicators.

### Prediction model performance

On the basis of these indicators, we built five ML models. Table 3 displays the results from the five ML models. We mainly compared the magnitude of the micro-F1 values. The micro-F1 value for each ML algorithm is the average of the five results from the 5-fold cross-validation. LightGBM has the best performance, with a micro-F1 value of 78.4%, significantly higher than the other four ML algorithms (77.7, 73.1, 72.4, and 71.6%, respectively).

**Table 3:** Comparison of five ML models.

**Model**	**Precision**	**Recall**	**F1-score**
RF	0.777	0.777	0.777
SVM	0.731	0.731	0.731
LightGBM	0.784	0.784	0.784
ANN	0.724	0.724	0.724
NB	0.716	0.716	0.716

**Figure 5:**
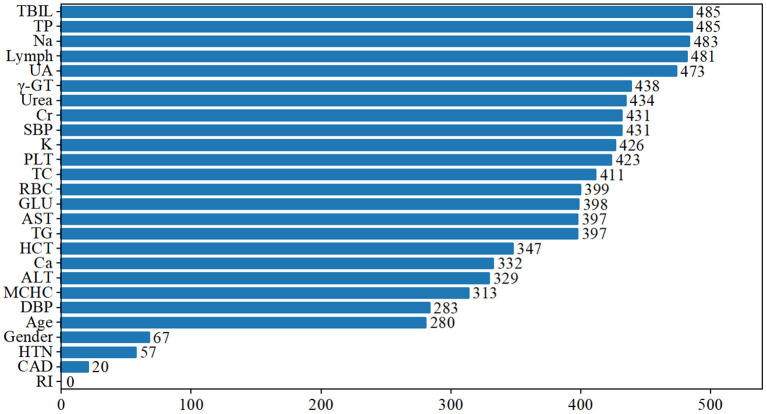
Ranking of feature importance in the LightGBM model.

We ranked the significance of all variables in the LightGBM model to comprehend the role of each better. The findings revealed that the five most critical characteristics of the model, TP, TBIL, Na, Lymph, and UA, contributed considerably to the prediction outcomes (Figure 5).

**Figure 6:**
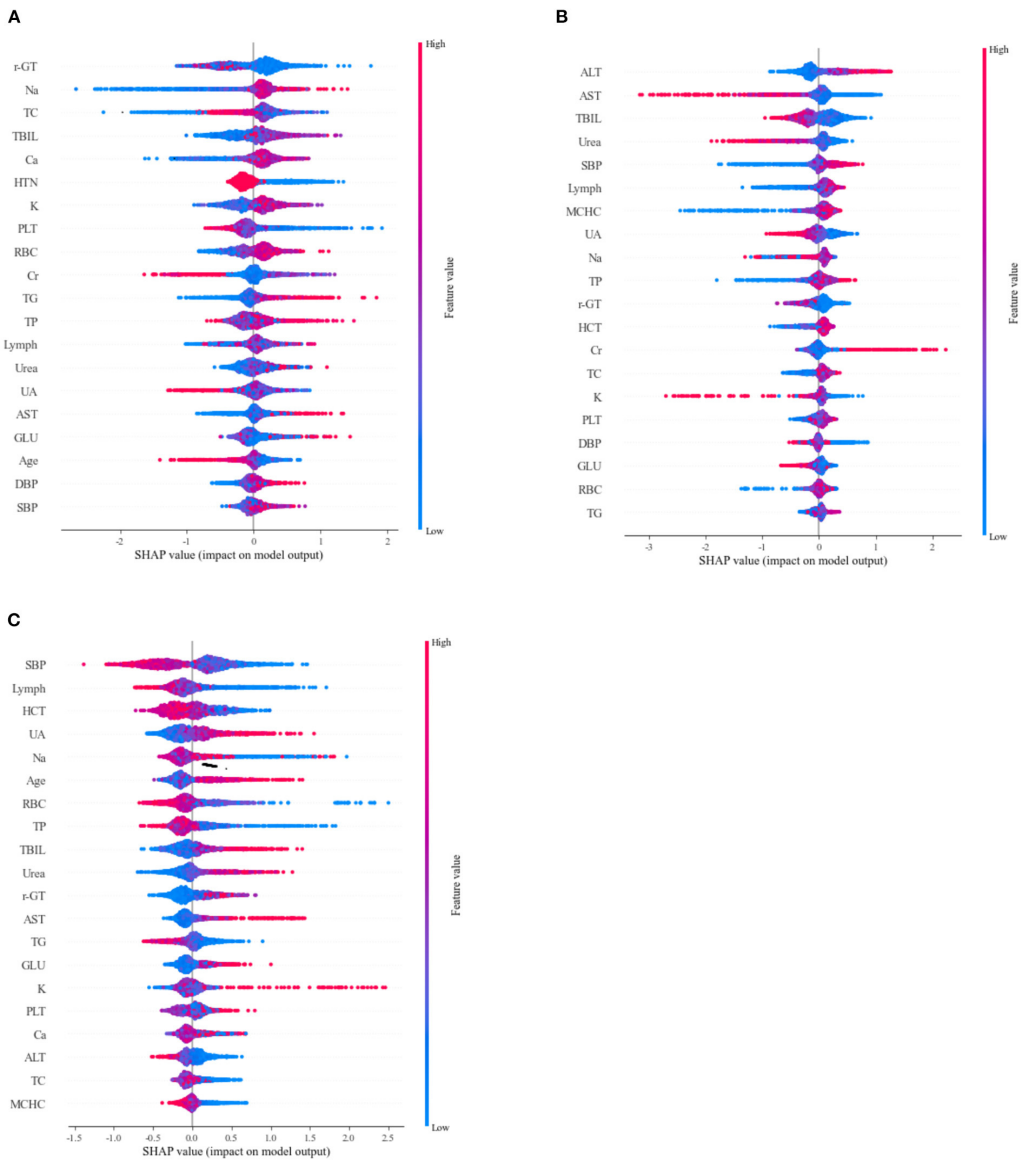
Sapropterin additivity interpretation (SHAP) scores were used to determine important features for predicting different drugs **(A)** Perindopril, **(B)** Amlodipine, and **(C)** Furosemide. Colors indicate whether the value of the feature is high (red) or low (blue).

We also figured out the saprophytic additivity explanation (SHAP) values of the LightGBM for different types of drugs to find the most important factors that drive model predictions. Figures 6A–C show the significant features of each class. Na, TBIL, and Ca increased, and γ-GT and TC decreased, favoring the classifier to predict Perindopril. Higher ALT and SBP, lower AST, TBIL, and Urea helped the classifier to predict Amlodipine. Higher levels of UA and lower levels of SBP, Lymph, HCT, and Na were beneficial for the classifier to predict Furosemide. Figure 6 depicts other critical characteristics of each medication class. We also visualized the interaction between features (Supplementary Figure S2).

## Discussion

Previous research has extensively discussed the significance of medication (24, 25). However, only about a third of persons with hypertension have it under control. The JNC study further supports the need for hypertension patients to get tailored treatment plans (26, 27). This study used SVM, RF, LightGBM, ANN, and NB algorithms to construct a prediction model combining patients' general information, history, and laboratory test findings. The proposed model performed well on the micro-F1 evaluation index and could assist clinicians in predicting medication regimens for elderly hypertension patients.

Amlodipine belongs to CCB, and several international, large-scale clinical investigations have proved its safety and efficacy in middle-aged and older populations. These studies include VALUE, ALLHAT, and ASCOTA (28–30). It also has a good effect in combination with other antihypertensive drugs. Furosemide, a diuretic that is both cheap and generally well-tolerated by patients, is a crucial tool for lowering blood pressure to normal in older adults with poorly controlled hypertension (31). Perindopril is an ACEI that reduces the risk of cardiovascular events and preserves target organs in elderly hypertensive individuals (32).

Hypertension generally causes kidney, heart, and other disease abnormalities, and antihypertensive medications may alter liver function. As a result, hypertensive patients usually need their blood routine, blood biochemistry, liver and kidney function, cardiac enzymes, and other items tested. Based on the literature reports (33, 34), clinical expertise, and data attenuation (more features, fewer patients fitting all parameters), we chose 22 blood test indicators that are somewhat associated with hypertension. In the blood routine, WBC is an important indicator of the inflammatory response, partly reflecting the control level of blood pressure in elderly hypertensive patients (35). Gagnon et al. (36) proposed that high HCT levels are related to potential risk factors for cardiovascular disease. Yang (34) found that RBC, Hb, HCT, MCHC, RDW-SD, and RDW-CV were all higher in the elderly hypertension group than in the control group (*P* < 0.05), suggesting that secondary erythrocytosis occurs in hypertensive patients and may induce an increase in blood viscosity. The MPV of the hypertensive group was lower than that of the control group (*P* < 0.05), indicating that evaluating PLT and platelet parameters might be employed as hypertension preventive and therapy detection indications in the elderly. Blood potassium, sodium, liver and kidney function, blood glucose, lipids, and other factors are all examined in blood biochemistry. If the test results show low blood potassium, secondary hypertension is possible. The detection of blood glucose and blood lipids may assist in determining if there are additional risk factors for cardiovascular and cerebrovascular disorders, and the evaluation of liver and kidney function is beneficial for physicians to pick antihypertensive drugs according to the patient's state. The results of these blood test indicators affect the choice of antihypertensive drugs and may guide the treatment of hypertension.

Despite the fact that there are defined clinical guidelines for the management of hypertension, there is still some confusion about how to treat hypertension. Therefore, we develop individualized medication by determining which characteristics influence drug selection. Our results showed that Age, SBP, DBP, Lymph, RBC, HCT, MCHC, PLT, AST, TBIL, Cr, UA, Urea, K, Na, Ga, TP, GLU, TC, TG, γ-GT, Gender, HTN, CAD, and RI were the typical indicators of the model. And among them, TP, TBIL, Na, Lymph, and UA were the five main indicators of LightGBM, which critically affected the selection of antihypertensive drugs. Wei et al. (37) discovered that the lower TP levels in elderly hypertensive patients were related to age, DBP, LDH, and HDL-C levels. So, monitoring TP is of great clinical reference value in the assessment and management of hypertension in the elderly. According to Wang et al. (38), UA and TBIL are essential markers of metabolic abnormalities in hypertensive patients, which are directly related to their development and can reflect the severity of hypertension. Kunutsor et al. (39) found that circulating TBIL levels were independently associated with an increased risk of cardiovascular disease. However, the relationship between TBIL levels in circulating blood and hypertension has not been elucidated. Numerous studies have shown that sodium in the diet influences blood pressure and that cutting down on salt helps bring blood pressure down (40–42). The INTERSALT research, a massive cross-sectional analysis, found that sodium was strongly linked to increased blood pressure with age (43). Guzik et al. proposed that hypertension enhanced T lymphocyte production in response to tumor necrosis factor-alpha and that T cells may provide a unique therapeutic target for hypertension therapy (44). At the same time, the ratio of neutrophils to lymphocytes is a special inflammatory indication that may be used to predict, treat, and forecast the course of primary hypertension by focusing on inflammation (45). The differences in indicators found between the groups helped direct our selection of antihypertensive drugs.

We compared five ML algorithms, and all were tested using a 5-fold cross-validation approach. In our study, the LightGBM model had a micro-F1 value of 78.4%, significantly higher than the other four ML models. These results demonstrate the superiority of the LightGBM model for determining which antihypertension drugs will be most effective in the elderly. LightGBM is an integrated model of decision tree for classification and regression prediction. It has an excellent version in disease diagnosis, such as Rufo (46) using LightGBM to predict diabetes, and Chen (47) proposed a weighted bagging-LightGBM model based on network embedding and PU learning for IncRNA disease association prediction. RF achieved great precision and micro-F1, 77.7%, behind only LightGBM. It is widely accepted that RF is the most useful integrated learning approach for both classification and regression (48). RF offers excellent classification accuracy, is tolerant to outliers and noise, and does not engage in overfitting (49). Both Subasi (50) and Singh (51) employed RF models to diagnose chronic renal disease and forecast cardiovascular disease, respectively. Besides, SVM, ANN, and NB are often applied in therapeutic situations. Shankar (52) developed an optimal multicore SVM model for feature-based thyroid illness classification. Radhimeenakshi (53) used SVM and ANN to classify coronary heart disease. The number of cases of coronavirus disease in China, Japan, Singapore, Iran, Italy, South Africa, and the United States in 2019 was predicted using an ANN model built by Nazar (54). Montazeri (55) created an NB-based prediction model to predict survival in various breast cancer types. To further explain the model, we also computed the SHAP values of the top-performing model, allowing us to examine the effect of the characteristics of each cause type on the model output.

Undoubtedly, the findings of this research have provided some groundwork for the following study. However, the current research does have a few limitations. This research is retrospective, and there is bias in the data, which is an issue that arises in all retrospective studies. On the other hand, the treatment of hypertension in clinical practice is mainly based on administering many medications in conjunction with one another. Only three kinds of single-drug modeling with greater frequency were chosen for the prediction investigation in this particular research, which is obviously not enough. In the following sections, we will discuss the multi-label classification, which may accurately forecast various treatment regimens.

## Conclusion

This study constructed a single-drug prediction model for antihypertensive drugs based on SVM, RF, LightGBM, ANN, and NB algorithms. And we also compared the efficacy of the model with evaluation metrics such as accuracy, recall, and micro-F1 to reduce the bias caused by a single algorithm and a single evaluation metric to some extent. The results showed that LightGBM predicted the best results compared with other algorithms, with a micro-F1 value of 78.4%. We also used feature importance ranking and SHAP values to enhance the interpretability of the model. We suggest that “increased Na, TBIL, and Ca, and decreased γ-GT and TC” can be prioritized for Perindopril, and “higher ALT and SBP, lower AST, TBIL, and Urea” can be prioritized with Amlodipine, “higher levels of UA, low levels of SBP, Lymph, HCT, and Na” can be prioritized for Furosemide. The study included patients from multiple medical institutions, and the study's results may help clinicians provide decision support in prescribing antihypertensive drugs to patients.

## Data availability statement

The raw data supporting the conclusions of this article will be made available by the authors, without undue reservation.

## Author contributions

TW and WZ contributed to the conception and drafting of the study. YY helped to develop new techniques and programs. Data analysis by TW, SX, JT, and CY. The final text was reviewed and approved by all writers.

## Funding

This study has been financially supported by the College of Medical Informatics, Chongqing Medical University, China, Student Research and Innovation Experiment Project (YJSZHYX202120).

## Conflict of interest

The authors declare that the research was conducted in the absence of any commercial or financial relationships that could be construed as a potential conflict of interest.

## Publisher's note

All claims expressed in this article are solely those of the authors and do not necessarily represent those of their affiliated organizations, or those of the publisher, the editors and the reviewers. Any product that may be evaluated in this article, or claim that may be made by its manufacturer, is not guaranteed or endorsed by the publisher.
