# Supplementary material for: A comparative study of antihypertensive drugs prediction models for the elderly based on machine learning algorithms
Source: Front Cardiovasc Med. 2022 Dec 1;9:1056263. doi: 10.3389/fcvm.2022.1056263 (PMC9753549; doi:10.3389/fcvm.2022.1056263)

**Supplementary information**

**Table S1 |** A summary of the selected medical features related to hypertension.

| Feature | Abbreviation | Normal reference (unit) |
| --- | --- | --- |
| systolic blood pressure | SBP | 90-139 mmHg |
| Diastole blood pressure | DBP | 60-89 mmHg |
| Glucose | GLU | 3.9-6.0 mmol/L |
| Triglyceride | TG | 0.45-1.69 mmol/L |
| Total cholesterol | TC | 3.1-5.7 mmol/L |
| Kalium | K | 3.5-5.5 mmol/L |
| Sodium | Na | 135-145 mmol/L |
| Calcium | Ca | 2.25-2.75 mmol/L |
| Urea | Urea | 3.1-7.1 mmol/L |
| Uric acid | UA | 50-420 μmol/L |
| Creatinine | Cr | 44-132 μmol/L |
| Alanine amiotransferase | ALT | 0-40 U/L |
| Aspartate aminotransferase | AST | 0-40 U/L |
| Total bilirubin | TBIL | 1.7-18.4 umol/L |
| Total protein | TP | 60-80 g/L |
| γ-glutamyl transpeptidase | γ-GT | 0-50 IU/L |
| Red blood cell | RBC | 4.0-5.5 10^12/L |
| Hematocrit | HCT | 0.35-0.45 L/L |
| Mean corpuscular volume | MCV | 80-100 fl |
| Mean corpuscular hemoglobin | MCH | 27-33 pg |
| Mean corpuscular hemoglobin concentration | MCHV | 320-360 g/L |
| Lymphocyte | Lymph | 0.8-4 10^9/L |
| Platelet | PLT | 100-300 10^9/L |
| Mean platelet volume | MPV | 6.3-10.1 fl |

**Table S2** | Counts of the five classes of antihypertensive drugs.

| Class | CCB | ARB | Diuretic | β-blocker | ACEI |
| --- | --- | --- | --- | --- | --- |
| Count | 23972 | 15968 | 15792 | 10452 | 4229 |

**Figure S1 |** Drug distribution of 36945 patients with hypertension.


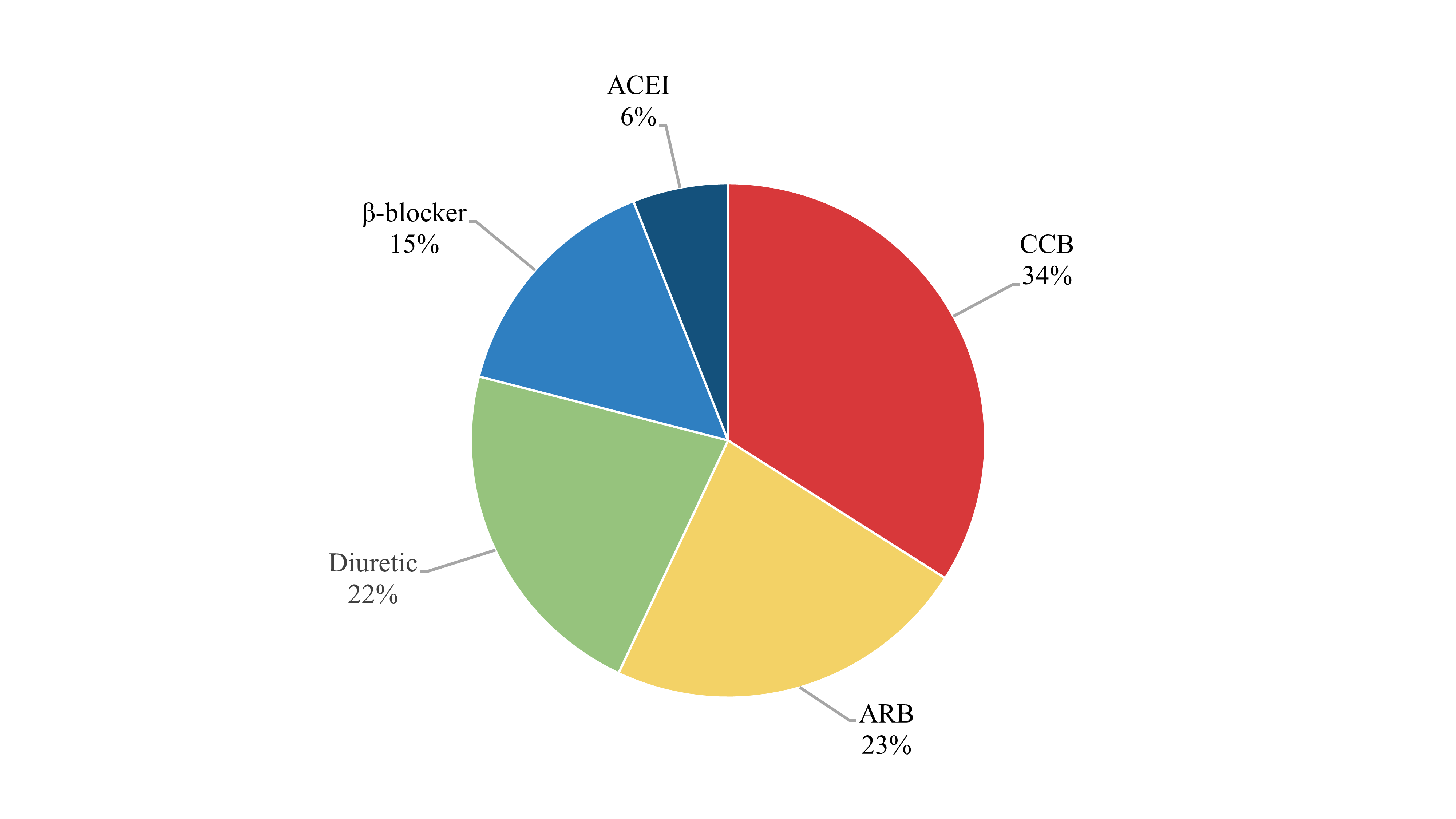


**Figure S1 |** Interaction between features.


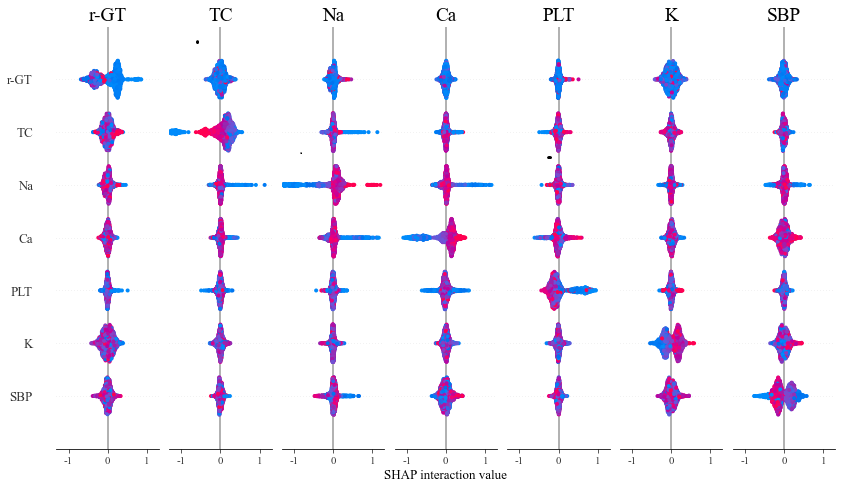

Supplement: Supplementary file 1 [file Data_Sheet_1.docx]
